# Adoption of a Tai Chi Intervention, Tai Ji Quan: Moving for Better Balance, for Fall Prevention by Rural Faith-Based Organizations, 2013–2014

**DOI:** 10.5888/pcd13.160083

**Published:** 2016-07-14

**Authors:** Dina L. Jones, Rachael W. Starcher, Jennifer L. Eicher, Sara Wilcox

**Affiliations:** Author Affiliations: Rachael W. Starcher, Jennifer L. Eicher, West Virginia University, Morgantown, West Virginia; Sara Wilcox, University of South Carolina, Columbia, South Carolina.

## Abstract

**Background:**

Translating evidence-based, community-delivered, fall-prevention exercise programs into new settings is a public health priority.

**Community Context:**

Older adults (aged ≥65 y) are at high risk for falls. We conducted a community engagement project in West Virginia to evaluate the adoption of a tai chi exercise program, Tai Ji Quan: Moving for Better Balance, by rural faith-based organizations (FBOs) and exercise instructors by recruiting 20 FBOs and 20 or more exercise instructors and by obtaining input from key stakeholders (representatives of FBOs, community representatives, exercise instructors) regarding potential barriers and facilitators to program adoption.

**Methods:**

We used both multistage, purposeful random sampling and snowball sampling to recruit FBOs and exercise instructors in 7 West Virginia counties. Two forums were held with stakeholders to identify barriers and facilitators to program adoption. We calculated separate adoption rates for organizations and exercise instructors.

**Outcome:**

It took up to 3 months to recruit each FBO with an adoption rate of 94%. We made 289 telephone calls, sent 193 emails and 215 letters, distributed brochures and flyers to 69 FBOs, held 118 meetings, and made 20 trips over a period of 31 days (8,933 miles traveled). Nineteen of 22 trained exercise instructors started classes, an instructor adoption rate of 86%. Key issues regarding adoption were the age requirement for participants, trust, education, and competing priorities.

**Interpretation:**

Although we had recruitment challenges, our adoption rates were similar to or higher than those reported in other studies, and the objectives of the community engagement project were met. Clustering the FBOs and having them located closer geographically to our location may have reduced our resource use, and using a recruitment coordinator from the local community may have enabled us to gain the trust of congregants and clergy support.

## Background

Falls are the leading cause of injuries in older adults (aged ≥65 y) (1). Participation in balance exercises, moderate-intensity muscle strengthening exercises, and moderate-intensity walking can reduce fall rates among this population by 30% (2). Thus, translating evidence-based physical activity programs for fall prevention into practice is a public health priority. The Tai Ji Quan: Moving for Better Balance (TJQMBB) tai chi exercise program is an evidence-based, community-delivered, fall-prevention intervention for older adults. There is a need for translation of the program into diverse community settings, such as faith-based organizations (FBOs).

## Community Context

Falls are a leading cause of death among older adults in West Virginia (3). West Virginia is the second most rural state in the United States and has the second oldest population (3,4). The prevalence of unintentional injuries is higher in older adults and in rural areas (3,5–7). Over 90% of West Virginia’s 55 counties contain medically underserved areas where older adults are at greater risk for falls (3,8–10). West Virginia also has high rates of physical inactivity and chronic conditions, both of which increase fall risk (11). Hence, fall-prevention interventions are needed in rural West Virginia. FBOs may be efficient, effective, and low-cost venues to deliver fall prevention interventions to rural older adults because of the cultural value rural adults place on religion, because of religion’s positive effect on health, and because church attendance increases with increasing age (12–14). FBOs are also one of the few institutions found in every rural area.

The overall objective of our project was to implement TJQMBB in FBOs in selected rural West Virginia counties and to evaluate adoption of this community engagement project by FBOs and exercise instructors. The adoption process involved 1) recruiting 20 FBOs, 2) recruiting 20 or more exercise instructors, and 3) obtaining input from key stakeholders (representatives of FBOs, community representatives, exercise instructors) on the potential barriers and facilitators to adopting TJQMBB. We considered each county and its FBOs as a community. The project was evaluated using the RE-AIM Framework (15).

## Methods

We began recruiting FBOs in May 2013. The TJQMBB intervention was delivered in 2 rounds (March to August 2014 and July to November 2014). In round 1, we identified 3 rural counties from among the state’s 55 counties that had a population density of fewer than 20 people per square mile, had high fall injury rates, and were close to West Virginia University in the northern part of the state. We intended to choose round 2 counties in the same manner; however, a colleague introduced us to an FBO in southern West Virginia, which led us to include that county and 2 bordering counties in the project. A seventh county was included in Round 2 because the pastor volunteered the FBO for the project. This project was approved by the West Virginia University institutional review board.

In Round 1, we purchased a mailing list containing 74 FBOs from www.infoUSA.com. Internet (n = 48), newspaper (n = 23), and Chamber of Commerce (n = 25) searches identified 96 additional FBOs for a total of 170. The list was sent to our university’s Extension Service agents in each county for verification. The list was next reviewed by 2 project partners, our faith-based consultant and members of our university’s Prevention Research Center Community Partnership Board, who added 18 and 6 more FBOs to the list, respectively. The list for Round 1 contained 194 FBOs, of which 30 were deemed inactive, leaving 164 FBOs in the sampling frame.

We made the intervention available to FBOs that could 1) secure space for the class, 2) identify an exercise instructor(s), 3) host at least one 16-week class, 4) recruit up to 15 congregation or community members for the class, and 5) who wanted to continue the class after the project concluded. Small FBOs were encouraged to partner with other FBOs or use community sites other than their church for the class.

In round 1 we used multistage, purposeful random sampling to select 50 FBOs (30%) from the sampling frame of 164 (16). We then purposively sampled 5 more FBOs to ensure diversity in denomination and size, which increased the list to 55 FBOs. A recruitment mailing was sent to these 55 FBOs in May 2013, which coincided with 3 rounds of press releases. A follow-up mailing was sent to nonresponding FBOs and FBOs not targeted in the initial mailing (n = 152). Because of a low response rate, we switched to a nonprobability sampling method in July 2013, snowball sampling, relying on networking and word-of-mouth to identify and gain access to the FBOs. We contacted various community constituents such as clergy, church congregants, health departments, fitness centers, ministerial associations, senior centers, civic leaders, and local news media (Table 1) (17,18). We also attended church services, prayer groups, ministerial association meetings, potluck dinners, and community festivals; volunteered at food pantries; and canvassed communities with brochures. We asked each person we spoke with to name other people we could approach about the project; these others were asked in turn to name other people we could approach and so on, in an ongoing process. We tracked the number of days, communications, meetings, trips, and miles traveled to recruit the FBOs. Our FBO adoption rate was the proportion of FBOs with trained instructors who started a class, multiplied by 100 (19).

**Table 1:** Characteristics of People Contacted (N = 218) to Recruit 17 Faith-Based Organizations by Using Snowball Sampling Methods, West Virginia, 2013–2014

Characteristic	N (%)^a^
Clergy and church offices	68 (31)
Congregants	45 (21)
Physical therapy and fitness centers	19 (9)
Civic leaders	17 (8)
Community members or organizations	16 (7)
Extension agents	12 (6)
Senior centers	11 (5)
Health clinics and departments	10 (5)
Ministerial associations	7 (3)
Faith-based organizations other than churches	6 (3)
Local news media	4 (2)
Other	3 (1)

a Percentages do not total 100 because of rounding.

The FBOs were asked to recruit instructors for exercise groups with help from the project staff as needed. We asked instructors to 1) attend 1 of 2 free, 2-day, TJQMBB trainings; 2) assist with participant recruitment; 3) teach at least one, 1-hour class twice weekly, for 16 weeks; 4) maintain program fidelity; and 5) continue class after the project concluded. Our instructor adoption rate was the proportion of trained instructors who started a class, multiplied by 100 (20).

Once all FBOs and instructors were recruited, a 5-hour forum was held with key stakeholders before each intervention round to identify barriers and facilitators to program adoption. The forums consisted of group discussions about the 8 stages of an intervention (21). Feedback from the forums was used to inform implementation of the intervention. Discussions were audio-recorded and moderated by the project leader who used a flipchart to record responses while an assistant took field notes. The assistant transcribed the recordings and assigned participant responses to the appropriate intervention stage. Responses were then added from the flipchart and field notes to produce the final document. The project leader reviewed all sources of data and the final document, discussed any discrepancies in stage assignment with the assistant, and summarized the results. At the end of each forum, the stakeholders completed a 10-item satisfaction survey. Survey items were rated on a 7-point scale ranging from 1 (strongly disagree or unproductive) to 7 (strongly agree or very productive). The survey evaluated the 1) forum location and schedule, 2) opportunity for taking ownership of the project, 3) stakeholder’s ability to network with other stakeholders, and 4) productivity of the discussions about the 8 stages of the intervention. Medians and interquartile ranges were calculated for the nonnormal data.

## Outcome

### Participating counties

The 7 project counties had a higher proportion of adults aged 65 years or older (17.8%) than the state (16.8%), but they were representative of the state population with respect to sex (50.6% female), race (96.1% white), and ethnicity (0.7% Hispanic) (22). The counties were diverse in size (range, 7,607–62,523 residents), industry (mining, manufacturing, and service), and fall rates (range, 5.03–6.97 falls/100,000 population) (22,23). Four counties (57.1%) were classified as low education (25% or more residents aged 25 to 64 years without a high school diploma or general equivalency diploma), 6 (86%) as low employment (fewer than 65% of residents aged 21 to 64 employed), and 2 (29%) with persistent poverty (20% or more of residents had incomes below the federal poverty level) (24). Six (86%) counties were ranked in the lower half of all state counties for health outcomes and had higher rates of physical inactivity and chronic diseases (ie, fall risk factors) compared with the United States overall (11,25). The most prevalent religious denomination among the FBOs was United Methodist.

### Recruiting FBOs 

From the letters sent to the initial 55 FBOs sampled, 3 (6%) FBOs responded and 43 (78%) did not respond; recruitment letters sent to 9 (16%) FBOs were undeliverable. One of the 3 responding FBOs enrolled in the project; the other 2 declined. Of the 152 FBOs that were sent the follow-up letter, 3 (2%) FBOs responded and 122 (80%) did not respond; recruitment letters sent to 27 (18%) FBOs were undeliverable. Two of the 3 responding FBOs enrolled in the project; the other one was too small to warrant training an instructor and holding a class and could not find another FBO to partner with. This recruitment strategy took 2.5 months to recruit 3 of the 20 FBOs.

Snowball sampling required 11 months to recruit the 17 remaining FBOs. We made 289 telephone calls, sent 193 emails and 215 letters, distributed brochures and flyers to 69 FBOs, and held 118 meetings. We made 20 trips over 31 days for a total of 8,933 miles traveled.

Eight of the FBOs were identified through word of mouth. Contacting clergy and church offices and congregants were the most successful methods of outreach. The number of days from first contact with an FBO to an FBO agreeing to join the project ranged from 1 to 280 days (mean, 62; SD, 65; median, 40). Excluding one FBO (an outlier) that took 280 days to recruit (the FBO joined and withdrew twice), it took between 1 and 119 days to recruit the FBOs (mean, 50; SD, 41; median, 35). The FBO denominations were Mainline Protestant, Evangelical Protestant, and multidenominational (Table 2). Two-thirds of the FBOs were small, with fewer than 100 members. Two of the 20 FBOs partnered with another, leaving 18 FBOs as the denominator for the FBO adoption rate. Two of the 18 FBOs withdrew (one because the instructor was ill and another because of competing projects). Sixteen FBOs had trained instructors; 15 of these began classes. The FBO adoption rate was 94%.

**Table 2:** Characteristics of 20 Faith-Based Organizations (FBOs) and 28 Exercise Instructors in Tai Ji Quan: Moving for Better Balance Program for Fall Prevention, West Virginia, 2013–2014

Characteristics	N
**Faith-based organizations (FBOs)**	
**How FBO was identified**	
Extension agent	1
Internet search	2
Ministerial association	1
Newspaper	1
Purchased mailing list	7
Word of mouth	8
**Outreach effort that resulted in FBO joining project **	
Clergy or church office	6
Congregant	5
Extension agent	1
Ministerial association	3
Physical therapy or fitness center	1
Recruitment mailing	3
Senior center	1
**Religious group and denomination**	
Protestant — Evangelical Church of God	1
Protestant — Evangelical Church of God in Christ	1
Protestant — Evangelical Nondenominational	1
Protestant — Evangelical Southern Baptist	1
Protestant — Evangelical Assemblies of God	1
Protestant — Mainline Episcopal	1
Protestant — Mainline American Baptist	5
Protestant — Mainline Presbyterian	3
Protestant — Mainline United Methodist Church	4
Protestant — Mainline Christian Church	1
Multidenominational	1
**Number of members (n = 15)^a^**	
1–99	10
100–199	2
200–499	3
500–999	0
≥1000	0
**Church members (n = 15)^a^^,^ b**	
1–99	12
100–199	2
200–499	0
500–999	0
≥1000	1
**Exercise instructors**	
**Relationship to congregation **	
Member	13
Active nonmember	1
Friend or family of member	2
Pastor	2
No formal connection	3
Other	3
Do not know	4
**Outreach effort that resulted in instructor joining the project**	
Church clergy or congregants	11
Community members	4
Newspaper article	1
Ministerial association	2
Volunteered	4
Family member or friend	1
Other class instructor	3
Extension agent	2

a Data were available for only 15 FBOs.

b The congregants who attended church services.

### Recruiting instructors

Twenty-eight of 38 people who were invited agreed to serve as an instructor. Ten declined, 3 because they did not have enough time, 4 for unknown reasons, 1 for philosophical disagreement with tai chi, 1 for health reasons, and 1 because of no access to an FBO. More than one-half of the instructors for whom we had data were members or pastors of their FBO (Table 2). The most common method for recruiting instructors was outreach by the church clergy or congregants (39%).

Six exercise instructors withdrew (3 for health reasons, 2 for unknown reasons, and 1 because the FBO withdrew), leaving 22 instructors to undergo training. We trained one instructor at 11 FBOs, 2 at 4 FBOs, and 3 at 1 FBO. After training, 3 instructors withdrew (2 for unknown reasons and 1 because the class didn’t have enough participants). Therefore, 19 of the 22 trained instructors started 16 classes, an adoption rate of 86%. Instructors ranged in age from 27 to 75 years (mean, 55 y; SD, 14.3 years). Of the 17 instructors for whom we had data, none had experience with tai chi, only 6 had ever led a group exercise class before, and only 3 had other exercise certifications (eg, Pilates).

### Stakeholder forums

Twenty-two stakeholders, 18 women and 4 men, attended 1 of the 2 forums that were held in November 2013 and April 2014. Eight attendees were FBO representatives and 14 were exercise instructors. Four attendees were also clergy. On the satisfaction survey, participants viewed the discussions as very productive and the networking opportunities as beneficial (all medians = 7, interquartile range = 1). The Figure lists barriers and facilitators identified by forum participants. For the planning phase, stakeholders recommended lowering the entry age for participants from 65 years or older to 55 years or older because some smaller FBOs did not have enough people over age 65 to make up a class, and younger participants could transport older ones to class. Offering FBO-sponsored classes was a barrier for some stakeholders with respect to recruiting participants in the larger community to take the class. They felt that people might be hesitant to attend activities at a church other than their own and suggested offering classes at more neutral sites, such as senior centers.

**Figure:**
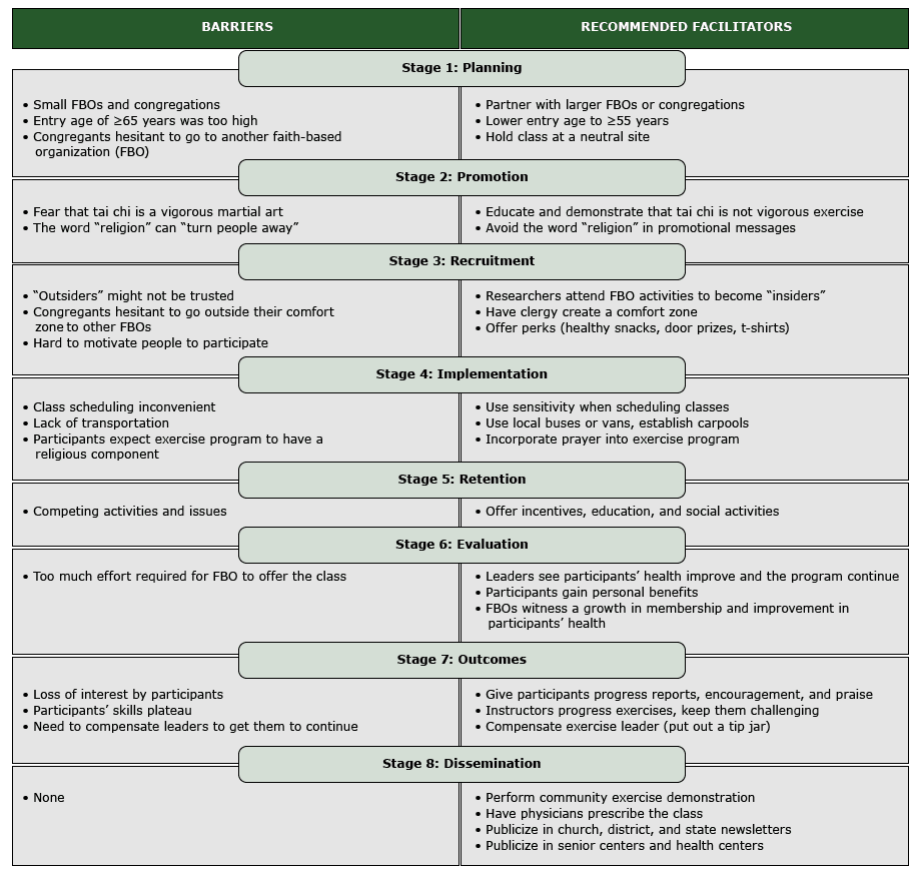
Key barriers and facilitators by intervention stage from stakeholders who attended project forums before adoption of the Ti Ji Quan: Moving for Better Balance program in rural faith-based organizations, West Virginia, 2013–2014.

Stakeholders suggested the need for education about what tai chi is and demonstration of the exercise in the promotion phase because some may fear the word tai chi and confuse it with more vigorous forms of martial arts. One suggestion was sending a video to FBOs to demonstrate that the program was not a high-intensity, high-impact martial art. 

Stakeholders identified distrust of outsiders (eg, nonmembers, researchers) as a major barrier for the recruitment phase. We were encouraged to attend church services and activities to become insiders. Stakeholders also felt that some participants might be uncomfortable traveling “outside of their comfort zone” (ie, their own FBOs) to other FBOs. Pastors could create a comfort zone by emphasizing the social bonding that could occur and by offering teaching and prayer in association with the exercise. In addition, those with the most need may be the hardest to motivate and thus, may need incentives to participate.

For the implementation phase, scheduling and transportation were identified as key barriers, as was participants’ expectation that the program would have a religious component. Stakeholders suggested using sensitivity when scheduling classes, for example, avoiding night classes and allowing participants to attend classes at other locations when needed. In addition, incorporating prayer into the exercise program may satisfy those expecting the program to have a religious component. The primary barrier identified for the retention phase was competing activities and issues such as other exercise programs, vacations, bible school, and family health issues. Recommended facilitators were sending class reminders, offering door prizes or gas gift cards, educating people about the program and holding potluck dinners.

Regarding the evaluation phase, stakeholders were asked “How would you measure success in this project?” They responded that instructors would measure success by the self-satisfaction they would feel in seeing participants’ health improve and the program continue after the initial project concluded. Participants would measure success through the physical, social, and spiritual benefits obtained from the exercise and by seeing the program grow. Finally, FBOs would gauge success by witnessing a growth in membership (ie, bringing more people into the church) and by seeing participants gaining health benefits. 

Regarding the outcomes phase, stakeholders were asked “What needs to happen for the program to continue after the project concludes?” Their responses focused on ensuring that participants remained interested and satisfied, that the instructors advanced the exercises so that the participants felt challenged, and that exercise instructors were satisfied and compensated. Finally, suggestions for disseminating the results of the project included hosting community exercise demonstrations, having physicians prescribe the class, and writing articles for publication on how to start a class.

## Interpretation

The objectives of the community engagement project were met. Several lessons were learned along the way that may inform future community engagement projects. We had to contact more FBOs than expected to reach our recruitment goals. Other studies used similar recruitment methods but did not have to contact as many FBOs. For instance, Lasater et al (26) recruited 20 of 31 churches in Rhode Island. The churches in that study, however, were larger (between 250 and 5,000 members) than the ones in our project, had a printed publication for communication (eg, newsletter), and were in middle-class urban areas. Our FBOs were mostly small, rural congregations with little or no formal means of communication and were in economically depressed areas (26). We also had to allow more time than anticipated for recruiting FBOs. The 3 months that we needed to recruit each FBO, however, was consistent with a study by Campbell et al (16) in which 3 to 6 months were required to recruit one church in rural North Carolina.

Switching from a scientific sampling process to snowball sampling allowed us to reach our recruitment goal; however, this approach was more costly in time and travel. We could have shortened our recruitment period had we planned to use snowball sampling from the outset. In addition, being from the university, we were considered outsiders, which was a disadvantage. Hiring a local resident as a recruitment coordinator may have helped overcome this barrier and reduced travel costs. We also could have saved time by limiting our project to the largest religious denominations in the state that had prominent state-level organizations; however, we wanted our FBOs to be as representative as possible of all FBOs in West Virginia. Researchers may want to factor in these issues when recruiting FBOs in rural areas.

Once FBOs were recruited, the FBO adoption rate for starting classes was high. On the basis of a previous study (19), we anticipated FBO adoption rates of between 52% and 80%. Our FBO adoption rate was higher than expected (94%), perhaps due to the small size of our FBOs, where information sharing may have been simpler and faster once the challenge of getting the information to the FBOs was overcome.

We worked closely with FBOs to recruit exercise instructors. It was challenging to find experienced instructors in rural areas, and as a result, only a few of our instructors had ever led group exercise classes. This highlights the need at the state or county level for a central repository that can identify exercise instructors who have been trained in evidence-based exercise programs. We experienced instructor attrition before and after the training sessions. Ideally, we would have trained at least 2 instructors per FBO, as recommended by Bopp et al (19); however, in some areas we had difficulty recruiting even one instructor, let alone 2. Therefore, researchers may want to plan to overrecruit instructors to account for attrition. Despite these difficulties, our instructor adoption rate was good at 86%, which was similar to the rate (83%) reported in a cardiovascular disease prevention program with aerobic exercise (20).

We struggled some with gaining clergy support, which has been shown to be essential for retaining faith-based partnerships (17). Our experience was consistent with results from a nationwide survey that reported a low level of involvement by faith leaders in action-oriented, health-related activities (eg, physical activity clubs, health screenings) (27). In that survey, faith leaders from rural or small congregations reported a lack of lay leadership and volunteers to organize activities as the most common barrier to implementing health and wellness activities (27). The FBOs in our project were mostly small, with few or no staff members, and had competing priorities in their communities, such as concerns over increasing rates of unemployment, substance abuse, and crime. Therefore, fall prevention and physical activity promotion were not high priorities. A lack of staffing and competing priorities may explain why, once clergy gave approval for the project, the exercise instructors and congregants became the program champions.

Hippolyte et al (17) used referral networks to recruit 5 FBOs in New York City by using motivational interviewing techniques. Those researchers had a stronger consistent presence at the FBOs in their study given the close proximity of the FBOs to the project staff. Thus, incorporating a behavior change strategy such as motivational interviewing, clustering the FBOs closer geographically, and using a recruitment coordinator from the local community may have reduced our resource use and provided an insider to gain more clergy support.

Some of the barriers (eg, lack of transportation, loss of interest, competing activities) and facilitators (eg, offering classes at convenient times, educating the community about tai chi) discussed at the stakeholder forums were consistent with those found in previous studies (28,29). A few stakeholders suggested that conducting classes at FBOs could be a barrier. One person commented, “She won’t come to my church; I won’t go to hers.” The stakeholders’ recommendation to hold classes at more neutral sites was in contrast to studies where churches were viewed as a facilitator for physical activity programs (28,29). Thus, we permitted FBOs to choose where to hold the classes. Furthermore, some of our FBOs were so small that the local senior centers were viewed as having more resources (eg, transportation) to support programming than rural churches, and partnering with them was encouraged.

Our project had several limitations. First, we were not able to ascertain why FBOs did not respond to our recruitment efforts, and consequently, we do not know if any FBOs had a philosophical disagreement with tai chi. Of all our interactions, only one person express a religious objection to tai chi. Second, our counties were of small to medium population size with residents who were older, unhealthier, and of lower socioeconomic status than most other US states. Thus, our results may not be generalizable to larger urban counties with better health profiles.

We successfully recruited FBOs and instructors for a translational study of a tai chi fall-prevention program in rural FBOs. We accomplished our objectives in a state that was ranked last in the nation for physical health, emotional health, and overall well-being (30,31). Translating evidence-based programs into practice in new settings, such as FBOs, in rural states is imperative to address critical health disparities.
