# Erratum, Vol. 13, July 14 Release

**DOI:** 10.5888/pcd13.160083e

**Published:** 2016-09-01

In the article “Adoption of a Tai Chi Intervention, Tai Ji Quan: Moving for Better Balance, for Fall Prevention by Rural Faith-Based Organizations, 2013–2014” the author inadvertently omitted a citation:

3. Li F, Harmer P, Fisher KJ, McAuley E, Chaumeton N, Eckstrom E, et al. Tai Chi and fall reductions in older adults: a randomized controlled trial. J Gerontol A Biol Sci Med Sci 2005;60(2):187–94.

The article was corrected on August 19, 2016, and appears online at http://www.cdc.gov/pcd/issues/2016/16_0083.htm. We regret any inconvenience this omission may have caused.

